# A very British state capitalism: Variegation, political connections and bailouts during the COVID-19 crisis

**DOI:** 10.1177/0308518X211072545

**Published:** 2022-02-14

**Authors:** Geoffrey T Wood, Enrico Onali, Anna Grosman, Zulfiquer Ali Haider

**Affiliations:** Western University, Canada; 3286 University of Exeter, UK; 5156 Loughborough University London, UK; Western University, Canada

**Keywords:** State capitalism, variegation, bailouts, political connections, crony capitalism, state capture, COVID-19, economic crisis, United Kingdom, comparative institutional theory, Liberal Market Economy

## Abstract

The COVID-19 pandemic has resulted in governments playing increasingly prominent roles as active economic agents. However, state capitalism does not necessarily serve broad developmental purposes, and rather can be directed to supporting sectional and private interests. As the literature on variegated capitalism alerts us, governments and other actors regularly devise fixes in response to a systemic crisis, but the focus, scale, and scope of the interventions vary considerably, according to the constellation of interests. Rapid progress with vaccines notwithstanding, the UK government's response to COVID-19 has been associated with much controversy, not only because of an extraordinarily high death rate, but also because of allegations of cronyism around the granting of government contracts and bailouts. We focus on the latter, investigating more closely who got bailed out. We find that badly affected sectors (e.g. hospitality, transportation) and larger employers were more likely to get bailouts. However, the latter also favored the politically influential and those who had run up debt profligately. Although, as with state capitalism, crony capitalism is most often associated with emerging markets, we conclude that the two have coalesced into a peculiarly British variety, but one that has some common features with other major liberal markets. This might suggest that the eco-systemic dominance of the latter is coming to an end, or, at the least, that this model is drifting towards one that assumes many of the features commonly associated with developing nations.

## Introduction

In October 2008, the UK government introduced a £500 billion rescue package to bail out financial institutions (BBC News, 2008), and it also nationalized some of them—such as Royal Bank of Scotland, by providing £20 billion against 63% of the equity. Ten years after the bailout, the government still owned 62% of the bank (Mor, 2018), and it is estimated that the bailout costed UK taxpayers £23.2 billion (Office for Budget Responsibility, 2018: Table 4.4). The financial crisis is but an example of how a Liberal Market Economy (LME^1^) such as the UK often does not operate according to market principles; indeed, a large body of scholarly literature highlights the divergence between neo-liberalism in theory and how it translates into practice (Choat, 2018; Hall, 2015). Moreover, the approach of the US, another LME, was somewhat different from that of the UK. For example, the US let Lehman Brothers collapse to prevent moral hazard by demonstrating that no financial institution is too-big-to-fail. In contrast, the UK government bailed out even relatively small banks, such as Northern Rock, which was nationalized in 2008. Even the form of intervention was rather different. The US government forced institutions receiving funding through the Troubled Asset Relief Program to be subject to stress tests. Injections of preferred equity under the Capital Purchase Program were provided with strings attached, although the former did not dilute the voting rights of common shareholders (Bayazitova and Shivdasani, 2012).

Such divergence in between the UK and the US is a function of the internally variegated nature of market liberalism rather than an anomaly (Mansfield, 2004; Jessop, 2011). State capitalism is when the state uses various tools for proactive intervention in economic production and the functioning of markets, including in the shape of share ownership, bailouts, and state financing of both state-owned and privately owned firms (Wright et al., 2021). In other words, the state assumes greater economic agency in response to events (ibid.). State capitalism leads to the expansion of the state's role as promoter, supervisor, and owner of capital, characterized by the growth of state-affiliated organizations and of statism of state–capital hybrids and of muscular forms of statism (Alami et al., 2021). While earlier literature on varieties of capitalism clearly differentiated between Coordinated Market Economies (CMEs), where the state was traditionally more connected to business and unionized labor, and LMEs (Hall and Soskice, 2001), it could be argued that it downplayed the potential for significant changes in state agency within a specific institutional regime (Wright et al., 2021). The emergence of new forms of state mediation is a characteristic of the variegated nature of British state capitalism, and in some respect, is not new. Moreover, in sustaining the market position of monopolies and oligopolistic firms providing utilities and outsourced state functions—and in bailing them out—the state is assuming the role of an *active economic agent*, but in the service of private, rather than public interests (Wood and Wright, 2015; Bracking, 2018). This highlights two important issues. Firstly, and as the literature on variegated capitalism reminds us, no national institutional order is pure, or dominated by markets or statism, but rather elements combine in serving a range of different purposes (Brenner et al., 2010; Jessop, 2011; Peck and Theodore, 2007; Dixon, 2011). Secondly, markets remain reliant—and arguably increasingly so—on the state even in supposedly lightly regulated contexts (Wright et al., 2021; Mansfield, 2004).

Other critics of the Varieties of Capitalism School have further challenged the assumption that LMEs are about light regulation of markets. For example, revolving door relationship between government and the private sector, and mutually reinforcing reward systems for senior managers, might suggest crony capitalism in the case of the US (Coates, 2014). As Gamble (2021) notes, part of the trajectory of post Brexit change has been to seek to bring the UK very much more in line with the US; whilst Brexit's proponents would pitch this in a positive light, it could be argued that the more negative features of the US model have become more pronounced. Again, to the dominance of rentiers from an early stage of British capitalism, and a lack of will and capacity to rejuvenate long-standing issues with manufacturing competitiveness, might be added an imperial-nostalgic hubris by policy makers; all this feeds into a lack of interest in complex policy solutions to support broad-based economic recovery and a focus on helping out those who are most influential (Coates, 2014). What this strand of literature alerts us to is the present fluidity in the UK situation, that market regulation is quite uneven, and that there is a recurrent pattern of insider interests capitalizing on a crisis to enhance their own position and worsening the internal distortions in the UK economy through doing this (Coates, 2014; Gamble, 2021). It is to such themes that this article speaks.

The COVID-19 pandemic, along with preceding and likely future shocks, was both predicted and willfully underprepared for, albeit that the scale of the pandemic was much greater in some economies than others; notably, the two largest and most archetypical LMEs, the US and the UK recorded the highest death rates among the mature economies. Yet, and as with almost all other economies, both governments were quick to abandon what may be conceived as the neo-liberal orthodoxy in extending large-scale bailouts across the national economy, a process that resulted in no little controversy owing to their scale and opacity (Giuliani, 2020). This leads to the question as to *what patterns are visible in terms of the bailouts, and what this might tell us about internal variegation in British capitalism*: does any unevenness in relief simply reflect necessary haste and the logical need of particularly hard-hit sectors, or does it reveal more about the operation of vested interests in the UK economy and the extent to which contemporary British manifestations of state capitalism represent the outcome of opportunistic rent-seeking behavior? What does this tell us about COVID-19 bailouts and capitalism more generally? Again, are the bailouts system-reinforcing, and represent continuities within British capitalism, or do they potentially represent both a response to, and a further manifestation of system-changing events, that will redefine the interplay between states and markets in the UK, with implications potentially for other types of capitalism? Should the British model of capitalism be theorized as state capitalist?

In this paper, we examine all the publicly listed firms in the UK, comparing those that received government financial support through various schemes during the COVID-19 pandemic relative to those that did not benefit from the government support, as the determinants of bailouts and their distributional characteristics are indicative of the variegation (e.g. the process of adaptation across scale and scope) within the British state capitalism. We rely on multiple sources of data, e.g., Bureau van Dijk's FAME, BoardEx, and Factiva, to obtain information about firm characteristics, political connections, and government bailouts, respectively. We find strong support for variegation in terms of sectors and other firm-level characteristics. Firms from the hospitality, transport, and support services sectors were most likely to be bailed out by the government. Larger business groups, labor-intensive or highly leveraged firms were also most likely to receive COVID-19 assistance. We also find strong evidence that politically connected firms had a higher likelihood to receive COVID-related financial assistance. Finally, firms with a majority shareholder had a higher likelihood of getting government financing which points out to shareholder concentration and preferential access of powerful actors to public finance.

The rest of the paper is structured as follows. In section “Theoretical context,” we discuss the theoretical context, based on the literature of variegated capitalism. Section “The 2008 and COVID-19 bailouts in the UK and around the world: What we know about who gets them” reviews the 2008 and COVID-19 bailouts in the UK and around the world. Section “Data” describes the data. Section “Methodology and results” offers the methodology and results. In section “Discussion and conclusion,” we provide a discussion and conclusion.

## Theoretical context

The undeniably heterogeneous literature on comparative capitalism has attained much influence in recent years in explaining why different national economies and associated firm practices progress on distinct trajectories, and, despite predictions by many of neoliberalism's proponents—and critics—convergence remains elusive (Meardi, 2018). The original *raison d’être* of the literature on comparative capitalism was to seek to make the case that the coordinated market model was at least as viable as the liberal market one, and, indeed, had a number of advantages in terms of social and physical infrastructure (Lincoln and Kalleberg, 1990; Dore, 2000; Hall and Soskice, 2001). Later work in this genre was much more skeptical as to the coordinated model's sustainability, and this seemed confirmed by the world eco-systemic dominance of market liberalism. The recovery in the confidence of the large European coordinated markets, and political crises and infrastructural decay in the two largest liberal markets would seem to confirm the original assumptions of comparative capitalism framework, rather than later critiques, developments, or departures. At the same time, trends became clear. Firstly, even as national models persist, there has been a general tendency (up until recently) to the partial dismantling of institutions with the aim of greater market efficiency (Peck and Theodore, 2007; Jessop, 2011). Secondly, production networks have become heavily internationalized, meaning that the consequences of specific patterns of practice in one model can spill over into another (Jessop, 2011; Peck and Theodore, 2007), whilst other work has highlighted how transnational investors might actively seek to undermine different national models (Harmes, 1998; Dixon, 2011). Finally, and, at least up until the excesses of Brexit and the Trump years, the LME model was widely upheld as the gold standard of capitalism (Wrenn, 2021). If capitalism was diverse, neoliberalism held eco-systemic dominance (Jessop, 2011).

However, none of this represented the triumph of unfettered markets. LMEs, which are defined as economies of the developed world marked by high levels of market competition (Hall, 2015) and characterized by a relatively minimalist state role where it provides the bare necessities for markets to operate such as the enforcement of property rights and the provision of essential public goods, have taken statist turns in the past, most notably the New Deal of the 1930s, post-war reconstruction, and the UK Labor government efforts at industrial policy in the 1970s. Another instance would be periodic waves of antitrust activity, even if in recent years, these instruments seem to have gotten rather rusty. Once more, in recent years the major LMEs had become quite statist in many respects, with burgeoning security and penal complexes, to supplement substantive military-industrial ones, and ecosystems of oligopolistic firms dependent on political patronage to operate outsourced state functions, and others (e.g. the oil and gas sector) ever more dependent on state subsidies (Wood and Wright, 2015; Erickson et al., 2020). Rather than no statism, the boundaries of what constitutes acceptable statism are somewhat fluid and defined in cultural and ideological terms; the loudest proponents of small government are often similarly vocal in calling for big militaries and the sustenance of favored industries (e.g. the Australian government's promotion of “fair dinkum” power, aka coal-fired power). This highlights a broader issue: although there is undeniable diversity in capitalism, no model is pure, with national systems exhibiting hybrid features (rather than exclusively statist or market-orientated) and impacting each other (Jessop, 2011; Peck and Theodore, 2007; Peck, 2019). The literature on variegated capitalism builds on such concerns and recognizes that decisive historical moments might adjust the internal configuration of systems and how they relate to each other (Boyer, 2021).

Meanwhile, theoretical work on internal diversity within varieties of capitalism draws on both traditional varieties of capitalism and the literature on variegated capitalism, to highlight the bounded nature of internal diversity within national systems, how on spatial and sectoral grounds, some actors may do much better out of the system than others, and how interests compete to ensure that at least parts of national systems are more to their liking (cf. Dore, 2006; Peck and Theodore, 2007). The literature on variegated capitalism highlights how internal contradictions and crises within national institutional orders result in spatially and temporally defined fixes. However, interventions are affected by the relative configuration of social, political, and economic forces, with some issues being prioritized and being treated as more important than others, with associated variations in the scale and scope of interventions (Jessop, 2011). Although it shares with the varieties of capitalism approach an interest in national level institutions, the variegated capitalism approach places more influence on the uneven and contested nature of internal variety within national institutional orders (Jessop, 2011).

The variegated nature of capitalism is particularly pronounced in the UK. After the Second World War, the government faced mass unemployment; the newly elected Labor government was highly interventionist, conducted a wave of nationalizations, bringing 20% of industry under state control, establishing the public National Health Service and other welfare schemes (Beer, 1965). In the following years (1960–1970s), the British approach was based on promoting domestic growth, based on efforts to liberalize domestic financial markets, with the financial center of the City of London an important source of international income, and an influential source of support for the Conservative party (Hall, 2015). The idea that the UK was being outcompeted and left behind was very strong in the late 1970s and early 1980s which coincided with broader angst of British (and the US) decline (Coates, 2000). The 1980s saw the beginning of neo-liberalism during which the role of the government evolved. Under Thatcher and subsequent governments, large-scale privatizations of state enterprises and services took place, including transport and utilities. However, despite high hopes, private contractors have not proved invariably better at managing government services than direct government supply. In some cases, the cost of otherwise providing government services was reduced, although often in tandem with some loss in quality; in others, prices soared and quality declined (Bayliss et al., 2020). Yet there were still episodes of forceful state intervention, especially to make labor markets more competitive (Gamble, 1994). The following Labor governments, most notably under Tony Blair, strengthened social welfare, but continued on the privatization path. By the end of the 1990s, the Triumph of the Anglo-American capitalism seemed complete, and neo-liberalism was unchallenged as the dominant ideology and had become inseparable from the discourse of globalization (Bishop and Payne, 2021; Gamble, 2006). These earlier shifts in the British model of capitalism, followed by more recent ones during the financial and COVID-19 crises highlighted in the next section illustrate the evolving role of the state and provide the case for the better conceptualization of the British model within the varieties of state capitalism framework.

This paper builds on three emerging concerns. Firstly, that no system is largely market-dominated, as economic relations are always embedded in complex social relations (Payne, 2006). As systems develop in response to external and internal crises, novel forms of state mediation may emerge (Wright et al., 2021), and this phenomenon has been quite visible in LMEs (Wood and Wright, 2015). Indeed, it could be argued that state capitalism has become an ever-more prominent feature; the state is increasingly assuming the role of an active economic agent, but in the form of working in concert with private interests to save the latter from their worst excesses, to support and sustain insider corporations, and in response to poorly prepared for (even if often widely predicted) events (Phan and Wood, 2020). Of course, the *raison d’être* of LMEs is to support private property owner interests, the assumption being that investors can make optimal decisions, leading to firm prosperity, growth, with society benefiting via the presumed trickle-down effect. However, one of the assumptions underlying this is that property owners are treated equally, without some receiving disproportionately favorable treatment over others.

Secondly, as the literature on variegated capitalism reminds us, there are “decisive moments of economic transformation and social restructuring” (Peck and Theodore, 2007: 763). Whilst the ultimate destination remains unclear, it is evident that the two largest LMEs have undergone changes, even if there remain important policy continuities. Whilst they were upheld as islands of constitutional stability and institutional maturity, both have seen the “political commanding heights captured by right-wing populism” (Cumming et al., 2020); and it remains unclear as to how temporary Trump's reverse will prove. With this, there have been serious drives to abandon democracy (the US) and open-ended institutional change (Brexit) (Cumming et al., 2020; O'Reilly et al., 2016; Sheppard, 2020).

In summary, this study is about novelty in the forms that states may take to act as economic agents in response to a crisis, how an LME may combine segments of market primacy with far-reaching interventionism, and the open-ended manner in which LMEs may be reshaped in response to said crisis. We look at the case of the UK, which along with the US, is largely considered to be close to the LME archetype (Clifton et al., 2013).

## The 2008 and COVID-19 bailouts in the UK and around the world: What we know about who gets them

Around the world, national governments have responded to the COVID-19 pandemic through bailouts to firms, especially in sectors deemed vulnerable or politically influential (Caldecott, 2020a; Abate et al., 2020; Muzio and Doh, 2020). Corporate bailouts in response to exogenous shocks (if any shock is truly so) are nothing new, but in the case of the US and UK bailouts, as noted above, their rapidity and opacity have raised particular concerns (Clifton et al., 2013).

**Figure 1.**
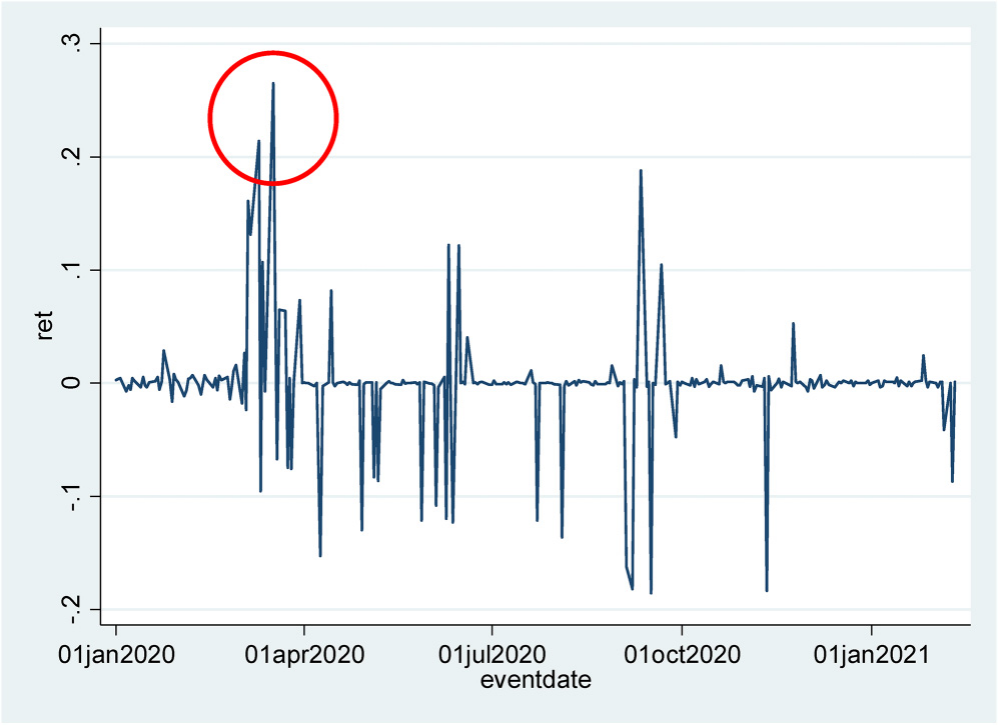
UK sovereign 5-year credit default swap rates. Notes: Source: EIKON Refinitiv, UK Government, 5-year credit default swap (CDS) rates denominated in GBP (RIC code: GBGV5YGBAB = R). Calculated as log return of daily average between the bid and ask, in basis points.

For instance, the rate for UK sovereign Credit Default Swaps (CDS) with 5-year maturity jumped from 18.58 to 24.22 basis points (around 27% log return) on March 17, 2020. On this day, Chancellor Rishi Sunak announced the first stimulus package (GBP 330 billion). As shown in Figure 1, the increase in the CDS rate was the largest one over the period from January 1, 2020, to February 10, 2021, suggesting that the fiscal stimulus was perceived to substantially increase sovereign default risk for the UK.^2^

The COVID-19 bailouts or assistance programs themselves were several and differed in structure and form to cater to the needs of different types of businesses affected by the pandemic. The retail, hospitality, and leisure sectors, for example, were provided with rates holiday for 2020 and 2021, and could also receive cash grants for up to GBP 25,000 per property. The Coronavirus Job Retention Scheme, on the other hand, was generally targeted towards affected businesses in all sectors to encourage them to keep employees on their payroll instead of laying them off; under this program, the government would cover a substantial portion of furloughed employees’ salaries (maximum of 80% or GBP 2500 per month as of October 2020). Programs were also designed with the aims of smaller and larger businesses in mind. The Small Business Grant Fund was linked to the properties owned by small businesses, while the COVID-19 Corporate Financing Facility, which was open to new applications until December 31, 2020, was more geared towards helping large companies with short-term financing needs. Businesses also had access to preferential financial terms through programs such as the Coronavirus Large Business Interruption Loan Scheme, the Bounce Back Loan Scheme, and Future Fund; the latter was particularly targeted towards innovative start-ups. Moreover, all VAT-registered UK companies were eligible for the Value Added Tax (VAT) deferral program (UK Government, 2021).

Although some areas of the bailout were obviously the most directly affected by COVID-19 (e.g. transportation and hospitality), a range of concerns emerged: these included whether firms that had recklessly accumulated large amounts of debt were disproportionately (both in terms of scale and selection) beneficiaries of relief; or whether they were firms with connections to the government officials or the ruling party. This raises the issue as to what patterns the bailouts assumed, and what can these patterns tell us about variegation in the state's role as an active economic agent in the contemporary UK? Secondly, although societies may revert to the normal state of matter after a period of crisis, a particularly large shock may result in fundamental redesigns in institutions and reconfigurations of practices. This raises the question of whether the form and focus of the bailouts suggest that they may have system-changing consequences. If the latter, the economic system may be more aligned with what the *régulation theory* tells us about state capitalism (Boyer, 2021).

The 2008 financial crisis led to a series of bailouts directed at the financial services sector, and more specifically, banks; opportunistic actions by banks in response to regulatory shortfalls placed entire national financial systems in jeopardy. Yet, the responses of governments varied according to institutional context (Cardillo et al., 2020). Although it has been argued that the bailouts were of net benefit to taxpayers (as the consequences of large-scale bankruptcies would have been much worse), bondholders of a handful of large investment banks benefited disproportionately (Veronesi and Zingales, 2010). In turn, this led to public disquiet that influential players were being rewarded for their recklessness, leading to demands for improvements in internal and contextual corporate governance (Cardillo et al., 2020; Onali et al., 2016). Again, it has been argued that the unintended consequences of the bailouts in undermining sovereign credit standing highlights the need for closer diligence and due caution (Acharya et al., 2014). Congleton (2009) notes that there was great heterogeneity in the response of governments around the world, highlighting the differences in the ability of institutions to mitigate and respond to the crisis.

Common to much of the literature is an assumption that an opportunity for institutional redesign and better regulation was squandered, through a combination of a lack of ability and capability (Acemoglu, 2009; Griffith-Jones et al., 2010). A further theme is the uneven or “muddling through” nature of responses both within and between nations, reflecting the contours of what is possible, influence, and autonomous choices (Jessop, 2014; Culpepper and Reinke, 2014; Münnich, 2016). The aftermath has led to public policies varying between moderating the post-crisis austerity and the spreading of the sphere of “authoritarian statism,” whereby a range of policy alternatives was simply removed off the table (Jessop, 2014). The latter does raise an important question: whilst state capitalism can potentially embody national development objectives, the limiting of the range of policy alternatives can mean that its sphere is circumscribed into domains that are placed beyond public scrutiny and debate.

The emerging body of literature on post-COVID bailouts has been quite different from the one on the earlier crisis. The former focuses on whether the bailout funds were allocated to the “right” companies, and whether governments are using or could use this opportunity to steer organizations in a better direction, especially a more environmentally responsible one (Steffen et al., 2020; Giuliani, 2020). In other words, governments may use their economic agency to promote greater “stakeholderism” (Gelter and Puaschunder, 2021). Caldecott (2020b) argues that there have been opportunities for national governments to entice companies to comply with the Paris climate change agreement, although there was uncertainty as to how this might best be done; indeed, within the US and the UK, there was a reluctance of government to make usage of this opportunity, with a significant number of those chosen for bailouts having poor environmental records. Indeed, Bowsher et al. (2020: 436) argue that the UK response was “fragmented and incoherent.” Further, there has been a broader pattern of government interventions to be uneven and slow, where political expediencies often overshadow evidence. In turn, multinationals may engage in arbitrage, profiting from imperfect global regulation and the economic interventions of different national governments. In practical terms, powerful economic entrepreneurs have the opportunity to remake national politics more to their own liking, again, capturing and directing state interventions in a manner that suits their own private interests (Giuliani, 2020). On the one hand, there were strong pressures on governments to be seen not to revert to “lemon socialism” where the public pays to bail out badly run firms (ibid.). On the other hand, the haste of the bailouts in the US and the UK raises the real question as to whether this ended up being the case.

There are a number of recent responses by the governments to crises where such competing tensions are observed. In looking at airline bailouts, Abate et al. (2020) found that national governments considered a range of different implications, ranging from relative national embeddedness to the provision of jobs and financial needs. Although this may lead to an increase in government ownership and control, it remains unclear as to what effects bailouts would have on the industry and, indeed, the environment (Abate et al., 2020). Whiteside (2021a, 2021b) similarly discussed the expansive role of the state in crisis management in what is considered as a liberal market economy—Canada—in the case of Air Canada. Having already received a $250 million bailout in 2009, nationalization was again a very likely outcome due to the airline's inability to sustain itself and lack of other viable rescue options. Oldfield (2020) argues that this may create numerous opportunities for corruption, given the involvement of many branches of government, the devolution of decision making, the allocation of funds outside of normal budgetary channels, and poor monitoring.

## Data

Our sample contains 1920 publicly listed UK firms. We collect financial and accounting data from Bureau van Dijk's FAME database over the 2010–2020 periods. We merge this database with BoardEx database for board-level political connections. BoardEx shows the connections of two organizations because of individuals with past or current tenure in both organizations.

Our dependent variable, *Government COVID Assistance*, is a dummy variable that is 1 if a firm has received any kind of COVID-related financial assistance from the governments of the UK or Ireland, including rates relief, tax deferral, job retention schemes, COVID Corporate Financing Facility, etc., and 0 otherwise. Even though the various assistance programs differ in nature (as highlighted in section “The 2008 and COVID-19 bailouts in the UK and around the world: What we know about who gets them”), they are all aimed towards bailing out firms that have been adversely affected due to the COVID-19 pandemic by providing financial assistance using taxpayer money. We searched for the relevant articles published in English in Factiva using a keywords strategy similar to Faccio et al. (2006) for the year 2020.^3^

Our key explanatory variable, *Political Connections*, is defined as a binary variable taking 1 where at least one board director of a firm is affiliated with the government, and 0 otherwise. We considered all cases where the connected organization is “Government,” whether it is the UK government (most cases) or a foreign government. The detailed definitions of other variables are given in Table 1.

**Table 1.** Description of variables.

Variable	Source	Definition
Government COVID-19 assistance	Factiva, Bank of England	Binary variable taking 1 if a firm has received any kind of COVID-related assistance from the governments of UK or Ireland, and 0 otherwise
Political connections	BoardEx	Binary variable taking 1 where at least one board director of a firm is affiliated with government, and 0 otherwise
Political connections—UK	BoardEx	Binary variable taking 1 where at least one board director of a firm is affiliated with the UK government, and 0 otherwise
Sector	FAME	Primary UK SIC (2007) codes, 2 digits
*Size*		
Firm size	FAME	ln(Total Assets + 0.001). Total Assets in thousands GBP
Companies in group	FAME	Companies in group (last available year)
*Value*		
Tobin's *Q*	FAME	Market-to-book ratio: Market capitalization/Total Assets
*Ownership*		
GUO–Direct %	FAME	Direct ownership percentage by global ultimate owner
*Capital structure and liquidity*		
Liquidity ratio	FAME	(Current Assets Stocks)/Current Liabilities
Interest cover	FAME	(Operating *P/L*)/Interest Expense
Gearing	FAME	(Non-current Liabilities + Loans)/Shareholders Funds

In Table 2, we compute the sectorial distribution of the UK public market. The largest category, in terms of a number of firms represented in our sample, is the financing industry (nearly a third of the sample), followed by manufacturing and professional and scientific services, each 12%.

**Table 2.** Sectorial distribution of the sample.

**Sector**	Observations	Percent
Agriculture	55	0.28
Mining	2,046	10.54
Manufacturing	2,299	11.85
Utilities	264	1.36
Construction	858	4.42
Retail	1,144	5.90
Transport	341	1.76
Hospitality	275	1.42
IT	1,661	8.56
Finance	5,885	30.33
Real estate	550	2.83
Professional & scientific services	2,233	11.51
Support services	1,056	5.44
Public administration & education	143	0.74
Health activities	209	1.08
Arts	308	1.59
Other services	77	0.40

Notes: Primary UK SIC (2007) codes, 2 digits. The SIC sector categories are recoded as follows: (1/3 = 1) (5/9 = 2) (10/33 = 3) (35/39 = 4) (41/43 = 5) (45/47 = 6) (49/53 = 7) (55/56 = 8) (58/63 = 9) (64/66 = 10) (68 = 11) (69/75 = 12) (77/82 = 13) (84/85 = 14) (86/88 = 15) (90/93 = 16) (94/99 = 17), where sector 1 is labeled “agriculture,” 2 “mining,” 3 “manufacturing,” 4 “utilities,” 5 “construction,” 6 “retail,” 7 “transport,” 8 “hospitality,” 9 “IT,” 10 “finance,” 11 “real estate,” 12 “professional and scientific services,” 13 “support services,” 14 “public administration and education,” 15 “health activities,” 16 “arts,” and 17 “other services.”

In Table 3, we show how our sample splits into four combinations according to government COVID-19 aid and political connections: for instance, about 14% of firms in our sample have a connection to the government AND received COVID-19 financial assistance from the government, which is slightly more than those firms that do not have a connection to the government (12%).

**Table 3.** Government COVID-19 assistance vs. political connections.

Political connections				
		No (obs/%)	Yes (obs/%)	Total (obs/%)
Gov COVID-19 Assistance	No (obs/%)	9,273(46%)	5,654(28%)	14,927 (74%)
	Yes (obs/%)	2,519(12%)	2,750(14%)	5,269(26%)
	Total (obs/%)	11,792 (58%)	8,404(42%)	20,196

Notes: Obs. represent firm-year observations, for 1,920 firms over the 2010–2020 period (panel data, strongly balanced). Government COVID-19 Assistance is a dummy variable that is 1 if a firm has received any kind of COVID-related financial assistance from the governments of the UK or Ireland, including rates relief, tax deferral, job retention scheme, COVID Corporate Financing Facility, etc., and 0 otherwise. Political connections measure is a dummy variable that is 1 if at least one board director of a firm is affiliated with a government-owned organization, and 0 otherwise.

Descriptive statistics are given in Table 4, and a correlation matrix of the variables is reported in Table A1 in the Appendix.

**Table 4.** Descriptive statistics for the key variables.

Variable	Obs.	Mean	Std. Dev.	Median
Gov COVID Assistance (1/0)	18,667	0.28	0.45	0.00
Political connections (1/0)	18,667	0.44	0.49	0.00
Political connections–UK (1/0)	18,667	0.29	0.45	0.00
Firm size (ln assets)	6,679	12.50	2.40	12.30
Gearing (%)	6,679	83.30	117.50	45.70
Tobin's *Q* (%)	6,679	1.30	2.50	0.80
GUO–direct (%)	6,679	98.90	7.50	100.00
Liquidity ratio (%)	6,679	1.80	3.60	1.10
Companies in group	6,679	94.00	199.00	28.00
Interest cover (%)	6,679	25.30	91.00	5.60

Notes: The statistics for the COVID aid and political connections variables are reported for the sample of Model (3), Table 5. The statistics for all other variables are reported for the sample of Model (4), Table 5.

## Methodology and results

We determine which types of firms received government COVID-19 assistance and which firms did not in two main ways. First, we conduct two-sample *t*-tests on the equality of means between the sample of firms that did not benefit from government COVID-19 aid, and the sample of firms that did (results available upon request). The means of the two samples are statistically significant on a number of firm attributes. First, firms that received COVID-19 assistance from the government are more often politically connected. Such firms are also significantly larger (by turnover), and are more labor-intensive in terms of the number of employees. They are surprisingly more profitable, measured by return on capital employed and return on assets. They are less knowledge-intensive, as measured by Research & Development.

Second, in Table 5, we estimate probit models^4^ with the dependent variable being 1 if the firm received government COVID-19 financial assistance, and 0 otherwise. We first test for sector fixed effects (Model 1). We then test for the influence of political connections alone on the likelihood of receiving government COVID-19 aid (Model 2), and whilst controlling for the sector fixed effects (Model 3). There is a significant likelihood that firms with political connections receive government COVID-19 aid.

**Table 5.** Which firms get COVID-19 aid: firm and sector characteristics, and political connections.

	(1)	(2)	(3)	(4)
	Gov COVID Assistance	Gov COVID Assistance	Gov COVID Assistance	Gov COVID Assistance
Political connections		0.346*	0.271*	0.186*
		(0.019)	(0.022)	(0.037)
Firm size (ln assets)				−0.081*
				(0.010)
Gearing				0.001*
				(0.000)
Tobin's *Q*				−0.000
				(0.007)
GUO–direct %				0.006*
				(0.002)
Liquidity ratio				−0.057*
				(0.022)
Companies in group				0.001*
				(0.000)
Interest cover				0.000
				(0.000)
Manufacturing	1.500*		1.446*	1.338*
	(0.053)		(0.053)	(0.079)
Utilities	0.454*		0.364*	0.594*
	(0.109)		(0.107)	(0.138)
Construction	1.351*		1.258*	1.286*
	(0.064)		(0.064)	(0.095)
Retail	1.998*		1.967*	1.907*
	(0.060)		(0.061)	(0.093)
Transport	2.227*		2.121*	2.022*
	(0.087)		(0.087)	(0.117)
Hospitality	2.446*		2.424*	2.507*
	(0.098)		(0.097)	(0.150)
IT	1.072*		1.050*	1.073*
	(0.056)		(0.056)	(0.087)
Finance	0.203*		0.152*	0.522*
	(0.052)		(0.053)	(0.090)
Real estate	0.963*		0.913*	0.954*
	(0.076)		(0.075)	(0.111)
Professional & scientific services	1.071*		1.041*	1.206*
	(0.054)		(0.054)	(0.086)
Support services	1.590*		1.577*	2.223*
	(0.061)		(0.061)	(0.100)
Public administration & education	0.868*		0.731*	0.984*
	(0.125)		(0.124)	(0.163)
Health activities	0.970*		0.924*	1.514*
	(0.104)		(0.102)	(0.162)
Arts	1.508*		1.487*	1.340*
	(0.087)		(0.089)	(0.162)
Other services	2.572*		2.504*	2.580*
	(0.189)		(0.196)	(0.280)
Mining	Base		Base	Base
Constant	−1.604*	−0.794*	−1.687*	−1.085*
	(0.046)	(0.013)	(0.047)	(0.259)
Observations	18,667	20,196	18,667	6,679

Notes: Probit models, the dependent variable is *Government COVID Assistance* defined as a binary variable taking 1 if a firm has received any kind of COVID-related assistance from the governments of the UK or Ireland, and 0 otherwise. Robust standard errors in parentheses. Description of variables is provided in Table 1. GUO is defined as direct ownership percentage by global ultimate owner. The base sector is set up to mining, which has the lowest probability of receiving a bailout, and all other sectors are compared to it. The variation in the number of observations is driven by measures of availability.

* denotes (*p* < 0.05). The main explanatory variable (political connections) is significant at the 0.01 level.

We then test the impact of political connections while controlling for firm-level characteristics, such as size, profitability, value, and capital structure (Model 4). Looking into marginal effects based on Model 4 in Table 5, firms with political connections have a 48% probability of receiving COVID-19 aid from the government, which is a higher likelihood relative to firms without political connections (42%).

Larger firms (by assets) are less likely to receive COVID-19 aid (model 4). Business groups, often multinationals, as measured by the number of companies in a group, are more likely to receive COVID-19 aid, and a few have indeed used it as an opportunity to get cheap short-term loans, while their contribution to the UK economy has been questionable. For instance, BASF, the German chemical group, received the biggest single payout (GBP 1 bn), via the Bank of England's COVID Corporate Financing Facility, while closing a UK plant and moving the work to France. Further, more than half the recipients of that scheme have cut jobs in the UK, including Japanese carmaker Nissan, US cruise company Royal Caribbean Group and Australian engineering company Worley.

We also find that the companies benefitting from the COVID-19 aid are experiencing liquidity issues. The government is actually assisting companies that are close to default, and may not have the ability to repay the loans.

To investigate the impact of belonging to a certain sector on the probability of receiving COVID-19 aid, we estimate the marginal effects related to the different sectors. Table A2 predicts the probability of different sectors receiving government assistance by holding all other variables constant at their means. COVID-19 has had a discriminatory impact upon different sectors (Donthu and Gustafsson, 2020), and in line with that, we see that certain sectors were more likely to receive aid. It is thus not surprising that the probability of receiving COVID-related assistance from the government is 88% among hospitality firms. For similar reasons, the corresponding probability for the transportation industry is also on the higher side, at 76%. Retailers were also highly likely to have received government assistance, particularly as many were forced to close temporarily or operate below capacity to meet the lockdown requirements of the government.

On the other hand, firms belonging to industries that have been adversely affected by the pandemic to a lesser extent had a lower likelihood of receiving help from the government. In contrast with the crisis of 2008, the financial industry had a low (20%) probability of receiving a government bailout this time. Other sectors such as IT, manufacturing, mining, arts, real estate, and public administration/education also had relatively low probabilities of less than 50%, which can be attributed to their stable demand during these times. Overall, these results highlight that firms belonging to sectors that were more adversely affected by the pandemic also had a better chance of receiving assistance from the government. State intervention and usage of taxpayer money thus appears to have been successful to that extent.

In further robustness tests (Table 6), we have defined the political connections variable more narrowly, to be as tightly associated as possible with the UK government. From the BoardEx database of over 20,000 affiliations with government, we have extracted those where the associated role of the board director in a UK firm contained one of the following words (which closely identified him/her with a UK government position): “CABINET,” “MINISTER,” “LORD,” “PARLIAMENT,” “SHADOW,” “GOVERNMENT,” or “STATE.” We then complemented this step by extracting all the firms that were affiliated with a government organization which contained in its name the following words: “UK” to identify the central government entities and “LONDON BOROUGH OF” to identify London Councils since they have their own budgets and capital allocation rights. This would identify such government entities as, e.g., “UK Cabinet Office,” “London Borough of Hackney,” etc. We created a dummy variable taking 1 if there was at least one board director with an affiliation to the UK government (as defined above), and 0 otherwise. Matching these data to the rest of the dataset of the UK publicly listed firms resulted in 26.82% of the sample taking 1 for UK political connections. The results based on this narrower definition of political connections remained robust and confirmed our previous results in the sense that there is strong empirical evidence that firms with connections to the UK government are more likely to obtain COVID-19 government aid.

**Table 6.** Which firms get government COVID-19 aid: Political connections to the UK government.

	(1)	(2)	(3)	(4)
	Gov COVID Assistance	Gov COVID Assistance	Gov COVID Assistance	Gov COVID Assistance
Connections to UK govt.		0.396*	0.313*	0.189*
		(0.021)	(0.023)	(0.036)
Firm size (ln assets)				−0.084*
				(0.010)
Gearing				0.001*
				(0.000)
Tobin's *Q*				−0.001
				(0.007)
GUO–direct %				0.007*
				(0.002)
Liquidity ratio				−0.057*
				(0.022)
Companies in group				0.001*
				(0.000)
Interest cover				0.000
				(0.000)
Manufacturing	1.500*		1.433*	1.339*
	(0.053)		(0.053)	(0.079)
Utilities	0.454*		0.337*	0.588*
	(0.109)		(0.106)	(0.137)
Construction	1.351*		1.243*	1.297*
	(0.064)		(0.064)	(0.095)
Retail	1.998*		1.929*	1.893*
	(0.060)		(0.061)	(0.092)
Transport	2.227*		2.128*	2.045*
	(0.087)		(0.088)	(0.117)
Hospitality	2.446*		2.405*	2.516*
	(0.098)		(0.097)	(0.149)
IT	1.072*		1.032*	1.070*
	(0.056)		(0.056)	(0.087)
Finance	0.203*		0.131*	0.524*
	(0.052)		(0.053)	(0.090)
Real estate	0.963*		0.894*	0.952*
	(0.076)		(0.076)	(0.111)
Professional & scientific services	1.071*		1.025*	1.200*
	(0.054)		(0.054)	(0.086)
Support services	1.590*		1.557*	2.213*
	(0.061)		(0.061)	(0.100)
Public administration & education	0.868*		0.691*	0.961*
	(0.125)		(0.122)	(0.163)
Health activities	0.970*		0.912*	1.503*
	(0.104)		(0.102)	(0.164)
Arts	1.508*		1.474*	1.340*
	(0.087)		(0.089)	(0.163)
Other services	2.572*		2.436*	2.552*
	(0.189)		(0.197)	(0.280)
Mining	Base		Base	Base
Constant	−1.604*	−0.759*	−1.643*	−1.070***
	(0.046)	(0.012)	(0.047)	(0.260)
Observations	18,667	20,196	18,667	6679

Notes: Probit models, the dependent variable is Government COVID Assistance defined as a binary variable taking 1 if a firm has received any kind of COVID-related assistance from the governments of the UK or Ireland, and 0 otherwise. Robust standard errors in parentheses. GUO is defined as direct ownership percentage by global ultimate owner. The base sector is set up to mining, which has the lowest probability of receiving a bailout, and all other sectors are compared to it. The variation in the number of observations is driven by measures of availability.

* denotes (*p* < 0.05). The main explanatory variable (political connections to the UK government) is significant at the 0.01 level.

We have also measured the likelihood of obtaining COVID-19 aid with the number of political connections to the UK Government per firm as an independent variable. Our results (available upon request) remain largely confirmed, meaning that more politicians on boards lead to a higher likelihood of receiving a bailout.

## Discussion and conclusion

This study highlights how the state may be bent if not to suit strictly market purposes, then definitely private ones. Five important findings help us to determine the variety of British state capitalism. First, the COVID-19 assistance was unevenly distributed across sectors. Transport, hospitality, and support services (such as renting and leasing, activities of employment placement agencies, tour operators, security, and office support) had the highest probability of receiving COVID financial assistance from the government. On the one hand, and, with the possible (or partial) exception of security, these groupings are areas particularly likely to be affected by the pandemic, and, hence, there would seemingly be a good prima facie case for a bailout. On the other hand, as will become apparent, the fruits of the bailout were unevenly spread within them. Again, all these areas are associated with insecure and precarious working, and, hence, the bailouts may have a post-COVID-19 effect of further skewing the composition of the labor market away from areas associated with relatively well-paid and secure work. This is not likely to reduce internal diversity or variegation within the economy, but rather sharpen internal divides and lead to further fragmentation (Dixon, 2011). Again, sector-focused bailouts may create precedents and expectations around what is deemed too important to fail, reinforcing the variegated nature of British state capitalism. Finally, such forms of bailouts are likely to have system-changing consequences in the sense that some sectors would become inadvertently predominantly state-owned or overburdened with debt they cannot repay.

Second, the government favored the more labor-intensive firms; these may have employed large numbers of workers (and voters), although there was no explicit intention to safeguard “good” jobs. Again, this would highlight the very partial and uneven nature of government intervention; COVID-19 relief did not seem to be deployed in any manner that might reset the economy on a more broadly-based better path. In any event, the eligibility criteria for the schemes were not explicitly set against smaller firms, as long as they generated revenues above the indicated threshold. This led some smaller trading companies owned by influential individuals to take advantage of the schemes, such as billionaire Sanjeev Gupta's metal trading businesses employing only 11 staff but securing £200 million through the Coronavirus Large Business Interruption Loan Scheme (Kleinman, 2021); concerns have been raised as to the perceived high probability of default. Gupta was previously controversial for lavishing gifts and hospitality on MPs, most notably Conservative MP, Nigel Adams (Collingridge, 2018).

Third, larger business groups were more likely to get the funding. This provides evidence for the existing arbitrage and self-dealing, highlights how powerful actors formed global alliances to take advantage of imperfect regulation, and the uneven effects of the British political system. Although an investigation of past donor activity by such firms falls beyond the scope of the study, it again highlights how some firms proved much more efficient in securing bailouts, and that ownership characteristics appear to matter a great deal. Earlier work on variegated capitalism highlights how the polymorphous forms assumed by national capitalism are bound up with political ties and what is contextually deemed appropriate (Hanieh, 2020).

Fourth, firms owned by powerful majority shareholders had a higher probability of receiving government COVID-19 aid, as did the politically connected firms. This finding suggests that firms can leverage their political capital to gain a competitive advantage, which in this case is the attainment of government aid (Barney, 1996). Viewed from an institutional standpoint, such political affiliations can increase the legitimacy (Suchman, 1995) of the firm in the eyes of the government: acting as an agent of the public, the government needs to ensure that the recipients of funds use them for their intended purposes; however, that is a difficult task given the time crunch presented by the pandemic and an ideal level of due diligence may not be possible to maintain. In such a situation, social networks between government members and leaders of the firms may substitute due diligence as sources of assurance. This may also be a manifestation of crony capitalism whereby public wealth is misappropriated by the government towards vested private interests that can potentially include both members of the firm and the government. This would again highlight the extent to which if state capitalism is the state serving as an active economic agent, then it does not mean that it necessarily serves public ends; indeed, the story of UK state capitalism is its focus on the servicing of private interest, and, the extent to which its ad hoc and somewhat capricious nature reinforces internal systemic diversity and variegation. A rich strand of literature on British capitalism (Coates, 2014; Gamble, 2021) highlights the manner in which insider interests have consistently managed to work the system to their own advantage, and, in turn, this has meant that serious efforts to resolve periodic crises rarely make progress. However, it is not to be taken that matters have remained the same: rather, Brexit and the populist turn, have imparted a further fluidity to the system that might favor the most predatory of interests.

An alternative explanation might be the information-superiority argument; in other words, those with good networks have superior information, and hence are more likely to submit strong applications for government relief schemes, albeit that our sample includes many SMEs who might be less able to process complex information or act upon it (Moro et al., 2015). However, in this instance, the explanation appears rather less plausible. Investigative accounts reveal an ongoing pattern of those with good political connections, but seemingly implausible qualifications or past commercial specializations getting lucrative government contracts (Iacobucci, 2020); interestingly, major recipients of Personal protective equipment contracts included a jeweler and a pest control company (Mahase, 2020). A very publicly criticized case of cronyism has been that of Greensill Capital, the collapsed finance company (under criminal investigation in Germany) which received access to the government emergency COVID-19 loans, primarily used for Sanjeev Gupta's business group of trading companies (also under investigation for fraud). Greensill paid the former UK Prime Minister, David Cameron, a compensation of more than $1 million a year for a part-time board role. As the pandemic unfolded, Cameron lobbied several ministers and civil servants on multiple occasions in an attempt to secure Greensill's access to government lending schemes. Greensill also employed another politician, former Australian foreign minister Julie Bishop as an adviser, paying her in excess of $600,000 (Smith and Pickard, 2021). More evidence emerged on how the COVID-19 loans were misused by Greensill, when the loan was given to Special Needs Group owned by a neighbor of founder Lex Greensill, after the two men jointly lobbied their local council to make use of Greensill's financial products (Smith, 2021). Of course, such evidence could be dismissed as anecdotal. Moreover, as evidence from emerging markets suggests (Aslund, 2019), there are a number of other ways cronyism might manifest, such as friends of friends winning government contracts, loans, etc. Accordingly, we also explored data related to obtaining government contracts during the COVID-19 crisis in additional tests, and our results remain robust; direct political connections do lead to more government support.

Fifth, there might be a systemic issue of overburdening with debt, as the firms with liquidity and solvency issues are more likely to be bailed out. The pharmaceutical industry alone was demanding for the government to waive the £370 million of government loans it was offered in 2020 to help meet additional costs arising from services to support the National Health Service, such as COVID-19 vaccinations, increased medication deliveries, and frontline care (Elgot, 2021). Clearly, this represents a temporary fix to problems that were ultimately structurally generated; the focus on value release rather than internal value generation (Lazonick and Shin, 2019). This raises the question of whether the LME model has increasingly shifted to one characterized by short-term fixes. Most countries have engaged in some or other form of COVID-related bailouts. However, in the UK this seems the latest in a series of interventions aimed at propping up the system through the regular reinflation of bubbles (cf. Lazonick and Shin, 2019), the timing of which is dictated by the events. At the same time, there is a growing body of evidence to suggest that the state and market (if the market is taken to mean the pursuit of private interests, whether by open competition or monopoly) should not be taken as opposite poles but can operate in concert.

This leads us to question what kind of variety British state capitalism is. There is, of course, no such thing as a pure market economy, and in the broadest sense, state capitalism, that is “the state assuming the role of an active economic agent” (Wright et al., 2021) can take many forms. The British state has assumed a very diverse role in the economy over the years, including the 1970s experiments in industrial policy and periodic later efforts at northern revival. State capitalism does represent an omnipresent phenomenon, but at times, it becomes more visible and extensive, as is the case presently in the UK (Dolfsma and Grosman, 2019). Nonetheless, our study suggests that whatever the characteristics of British state capitalism in the past, recent events would suggest that it has assumed at least some of the features of crony capitalism, as commonly understood in the literature. This raises the issue as to why? And are they there to remain after the crisis? Or are they temporary adjustments that were necessary reactions to accelerate the process of recovery, where the government formed alliances with powerful actors in response to these pressures?

For many years, scholars have drawn a comforting distinction between emerging and mature markets, yet rather than the former changing and the latter associated with fundamental stabilities, this is no longer very clear-cut. What is evident is that in LMEs, vested interests are increasingly open in seeking political solutions to their economic problems, and that capture of at least segments of the state has both reinforced internal variegation and made it harder to secure systemic stability. A caveat is in order here. State involvement does not necessarily entail more corruption; again, corruption assumes many forms, and cronyism, that is, the leveraging of personal networks and informal agreements is different to, say, bribe-taking. However, what this study does demonstrate is that a small group is able, through their position and ties, to secure preferential access to public assets, which would broadly fall within the generally accepted definition of crony capitalism (Singh and Zammit, 2006). Bishop and Payne (2019) highlight the extent to which the decay of the neo-liberal orthodoxy has brought a much wider range of issues into play, and challenges how we understand politics and society. Arguably, this might include a reassessment of what we understand about corruption, and how mature democracies might become more corrupt.

Therefore, the contributions of our study are threefold. First, we contribute to the literature on varieties of capitalism and variegations of capitalism, by focusing on the changing role of the state in the UK capitalism. To the varieties of capitalism literature, we contribute a more nuanced view on LMEs especially in times of crises, when state interventions take center stage, a variety of tools, and have long-lasting structural consequences. Although this literature has accorded increasing attention to institutional change, rather less attention has been accorded to whether liberal markets have significantly changed since the taxonomies were derived in the late 1990s. Again, whilst Hall and Soskice's (2001) approach centered on the role of economic actors, more attention was accorded to the firm rather than state economic agency. To the literature on variegated capitalism, we advance the notion that the government can adopt an uneven approach, with varieties of scale and scope, depending on the orientation of interests, sectorial dynamics, choices to augment support, and self-dealing interests.

This leads us to our second contribution, to the political connections and crony capitalism (cronyism) literature, as our study provides robust empirical evidence for the manifestations of cronyism in the UK in the context of allocation of government funds to assist the firms during the COVID-19 pandemic. We observe a higher likelihood of getting state COVID-19 aid for firms with politicians on boards. This also brings us to a fundamental rethink of cronyism in the context of a liberal market economy and bailouts, which would not have usually been thought of as conducive to such overt forms of corruption; the international business literature, for example, commonly assumes that corruption is something experienced by liberal market firms when they venture abroad. Finally, from an empirical point of view, we collected a unique dataset on COVID-related bailouts in the UK, which has not yet been explored in the literature, tested on an empirical specification which allows us to delineate in a causal way the characteristics of firms which led to the higher likelihood of success in getting the state COVID-19 aid.

It might be interesting to investigate to what extent the COVID-19 crisis was an opportunity for the LMEs to respond to the increasing globalization concerns by refocusing on the national economies in their fight against the virus which was precisely facilitated by globalization (Sheppard, 2020); in other words, whether cronyism may nonetheless lead to the emergence of new national champions or simply further grift. Clearly, the LME model is drifting away from what was commonly depicted as institutional maturity in the early 2000s (Hall and Soskice, 2001), to one associated with systemic fixes that are increasingly capricious in favoring some interests over others. If statism never really went away, British state capitalism—that is, the state playing the role of an active economic agent—has both extended its scale and scope, and narrowed in a manner that would be more reminiscent of the type of crony capitalism traditionally associated with emerging markets.

Finally, there is a large body of work on crony capitalism and state capture focused on so-called emerging markets (Aslund, 2019; Pei, 2017; Reinsberg et al., 2021); it can be argued that this is increasingly relevant to understanding happenings in LMEs, rather than applying the features of the later model as a benchmark against which emerging markets should be judged. State capitalism is clearly ubiquitous, but it plays out in many ways that illustrate the tensions and contradictions between general trends in the world system, uneven outcomes in space and scope, and between ad hoc fixes and efforts to set national economies on more stable paths. Again, the present COVID-19 interventions should be seen in terms of a longer pattern; ad hoc state interventions may both help sustain an existing systemic order and also drive its further fragmentation. This study is broad in terms of including in its scope all government packages designed towards helping out businesses affected by the pandemic. Future research can pursue a narrower purview by not only focusing on specific program clusters (e.g. those that are targeted towards a particular category of businesses or those that provide short-term loans), but also comparing the efficacy of different schemes within and between those clusters.

## Notes

1. In the influential 2001 *Varieties of Capitalism* collection, Hall and Soskice (2001) define liberal market economies as characterized by, inter alia shareholder primacy, arm's length contracting, thinner associational ties and lighter regulation of markets when compared to the Coordinated Markets of Scandinavia, the Rhineland and Japan.

2. CDS contracts provide compensation to the investor if the related entity (in this case, the UK) defaults on its obligations, and the CDS rate is similar to the premium of a typical insurance contract: a higher CDS rate indicates that the probability of default of bonds issued by the UK government has increased.

3. We have used the following keywords strategy to search for relevant articles: GOVERNMENT or STATE and [BAILOUT or CORPORATE BOND PURCHASE or BANK OF ENGLAND or COVID-19 FACILITY or COVID-19 or COVID 19 or CORONAVIRUS or COVID CORPORATE FINANCING FACILITY or CREDIT LINE or NATIONALISATION or DEBT or FUND or BAILOUT or BAILED OUT or RESCUE or RESCUE PACKAGE or INJECTION or RESTRUCTURE or RESTRUCTUR* or AID].

4. Our results are robust to alternative specifications, such as logit.

## Appendix

[Table table7-0308518X211072545] [Table table8-0308518X211072545]

**Table A1. table7-0308518X211072545:** Pairwise correlations for the key variables.

Variables	(1)	(2)	(3)	(4)	(5)	(6)	(7)	(8)	(9)	(10)	(11)
(1) Gov COVID assistance	1.00										
(2) Political connections	0.13*	1.00									
(3) Pol connections – UK	0.14*	0.73*	1.00								
(4) Firm size (ln assets)	0.11*	0.35*	0.37*	1.00							
(5) Gearing	0.11*	0.09*	0.10*	0.23*	1.00						
(6) Tobin Q	−0.01	−0.01	−0.01	−0.06*	−0.09*	1.00					
(7) GUO direct	0.04*	0.07*	0.03*	0.05*	−0.01	0.00	1.00				
(8) Liquidity ratio	−0.18*	−0.08*	−0.08*	−0.12*	−0.13*	0.00	−0.01	1.00			
(9) No of cos in group	0.09*	0.12*	0.16*	0.46*	0.15*	−0.01	0.04*	−0.09*	1.00		
(10) Interest cover	−0.02*	0.03*	0.02*	0.00	−0.15*	0.10*	0.01	0.07*	−0.05*	1.00	
(11) Sector	−0.02*	0.00	0.02*	−0.01	−0.02*	−0.01	−0.04*	0.08*	0.02*	0.06*	1.00

Notes: Significance levels: * for a *p*-value of 0.05 or less.

**Table A2. table8-0308518X211072545:** Sector-wise likelihood of receiving government COVID-related assistance.

Sector	Gov COVID aid
Mining	0.089*
	(0.011)
Manufacturing	0.496*
	(0.014)
Utilities	0.223*
	(0.035)
Construction	0.479*
	(0.025)
Retail	0.707*
	(0.020)
Transport	0.757*
	(0.029)
Hospitality	0.879*
	(0.026)
IT	0.390*
	(0.020)
Finance	0.205*
	(0.015)
Real estate	0.346*
	(0.031)
Professional & scientific services	0.441*
	(0.019)
Support services	0.806*
	(0.020)
Public administration_& education	0.349*
	(0.054)
Health activities	0.561*
	(0.058)
Arts	0.496*
	(0.059)
Other services	0.886*
	(0.052)
Observations	6679

Notes: Marginal effects for each sector are reported on the basis of model 4 of Table 5. Standard errors in parentheses. All predictors at their mean value. * *p* < 0.05.
